# Regeneration Mechanism of Sulfur Absorption Via Samarium-doped Cerium Adsorbents in the Gas Atmosphere of O_2_/N_2_

**DOI:** 10.3390/ma13051225

**Published:** 2020-03-09

**Authors:** Xuechao Hu, Junhui Dong

**Affiliations:** 1 School of Materials Science and Engineering, Inner Mongolia University of Technology, Aimin Street 49, Hohhot 010051, China; huxuechao777@126.com; 2 Inner Mongolia Power Research Institute, Hohhot 010020, China; 3 Inner Mongolia Key Laboratory of Graphite and Graphene for Energy Storage and Coating, Aimin Street 49, Hohhot 010051, China

**Keywords:** adsorbent, SDC, desulfurization, sulfur capacity, regeneration

## Abstract

Sulfides existing in many high-temperature gas mixtures have a negative effect on various industrial applications. Ce-based adsorbents are becoming a hotspot in the high-temperature desulfurization process owing to their excellent thermal stability at high temperatures and regeneration capacity. In this study, we investigate the regeneration path of samarium-doped cerium (SDC) sorbent at high temperature. The SDC adsorbent showed a good sulfur removal ability and excellent regeneration capacity. Ce_2_O_2_S and Ce(SO_4_)_2_ are observed in the used adsorbent, and Ce_2_O_2_S is the main sulfur-containing species. The regeneration path of the Ce_2_O_2_S is the key to the regeneration mechanism of the adsorbent. There are two regeneration paths for the Ce_2_O_2_S at high temperature in O_2_/N_2_ gas mixture. In air stream, the Ce_2_O_2_S is oxidized to Ce_2_O_2_SO_4_ and then decomposes into CeO_2_ and SO_2_. In a 2% O_2_/N_2_ gas condition, the Ce_2_O_2_S directly generates CeO_2_ and elemental sulfur with O_2_ assistance.

## 1. Introduction

With the rapid development of human society and fossil-fuel consumption increasing, serious environmental problems are gradually becoming significantly conflicted with economic development. Coal is one of the most sufficient fossil energy resources with inexpensive cost, and the consumption of coal has accounted for over 20% of consumed fossil fuels in the world [1,2]. Integrated gasification combined cycle (IGCC) power-plant is one of the strategies for efficient utilization of the coal. In the IGCC system, coal is first converted to CO and H_2_ (synthesis gas) and then further reformed to H_2_ and CO_2_ via the water gas shift reaction [3,4]. In this process, synthesis gas can be considered a raw feedstock to produce the value-added chemicals and fuels, and H_2_ as a high-density energy is used for producing energy. However, elemental sulfur in the coal is converted into hydrogen sulfide, and the presence of hydrogen sulfide seriously corrodes subsequent systems piping and catalytic systems. Therefore, it must be removed after the hot coal gas generation.

The H_2_S mixed in the high-temperature gas is often cleaned by a conventional amine solution method at a lower temperature (i.e., cold coal gas desulfurization) [5]. The operating temperature of the amine solution is below 150 °C. Thus, it will result in additional thermosteresis due to the cooling and heating process. Another method is to adsorb H_2_S by solid sorbents at high temperature (i.e., hot-gas desulfurization (HGD)) [6,7]. Therefore, HGD process (650~900 °C) is an efficient method for hot syngas purification. The overall thermal efficiency and power efficiency of the IGCC system will increase about 3% when using the HGD process [8]. Therefore, the HGD process (650~900 °C) is an effective method for hot synthesis gas purification. Investigating suitable adsorbents for desulfurization is necessary for the HGD process. Many kinds of metal oxides, based on the metals Ba, Ca, Co, Cu, Fe, Mn, Sr, W, and Zn, exhibit high potential for desulfurization at high temperature [9,10,11,12,13,14,15,16]. However, some metal oxides are generally degenerated in a reducing gas mixture during HGD process due to thermal instability at high temperature, such as Zn evaporation and Cu-oxides reduction [17,18]. For Mn-based adsorbents, a high concentration of desulfurization equilibrium is a main disadvantage [19]. The main problem of the Fe-based desulfurizer is that the desulfurization precision is not high, resulting in a large amount of reaction heat loss during the sulfur removal process [20]. For Ca-based sorbents, the formation of CaSO_4_ from used sorbents inhibits oxygen diffusion and hence suppresses the regeneration process, so there is not full regeneration by the air [21].

Regeneration products of sorbents are another important factor that should be considered for the HGD process. Generally, sulfur dioxide is a main product during the sorbent regeneration process, which requires additional decontamination. Compared with sulfur oxide, elemental S is a desired regenerated product. This could not only simplify the desulfurization procedure but also possesses a marketable value. Therefore, in comparison with the above-mentioned metal oxides, the Ce-based sorbents are becoming a hotspot in the HGD process. The CeO_2_ possesses excellent thermal stability and doping some elements can improve the CeO_2_ microstructure (e.g., surface area, pore size) and redox behavior as well as desulfurization activity [22,23,24,25,26,27,28]. Most of all, production of elemental sulfur can be relatively obtained during the regeneration process of the Ce-based adsorbent [29]. The detailed performance of the Ce_0.8_Sm_0.2_O_1+δ_ (SDC) sorbent in different conditions (e.g., temperature, H_2_, CO, and H_2_S concentration) and the SDC microstructure have been investigated in detail in our previous work. Therefore, in this study, we focus on the regeneration mechanism of the SDC sorbent and accurately display two different regenerated paths of the SDC sorbent at different O_2_/N_2_ gas conditions. 

## 2. Experimental

### 2.1. Preparation of the Sorbents

(SDC) sorbent was synthesized through a coprecipitation method [30] described as follows. Stoichiometric chemicals (Ce(NO_3_)_3_·6H_2_O, Sm(NO_3_)_3_·6H_2_O) were mixed in appropriate deionized water solution with continuous stirring. The precipitant of oxalate acid solution (1 M) was then added into the above mixed solution to form white precipitate. The precipitate is the SDC precursor, which was then cleaned by distilled water for three times and then washed two times in ethanol. The obtained precipitates were dried at 80 °C in the drying oven overnight. The final SDC precursor was sintered at 800 °C for 2 h to get the SDC sorbents. CeO_2_ powder was also prepared by the coprecipitation method. 

### 2.2. Preparation of the Ce-O-S Powder and Corresponding Regenerated Powders

Ce_2_O_2_S-containing powder was prepared by the desulfurization process experiment. 500 mg sorbent powder was put into a quartz tube flowing 3000 mg/m^3^ H_2_S—10% H_2_—20% CO-N_2_ balance (volume ratio) gas mixture at 800 °C. After the appearance of the breakthrough point, the final Ce_2_O_2_S-containing powder could be obtained, marked as Ce-O-S powder. The Ce-O-S powders were directly calcined at 800 °C in the air for 6 h to form the Re1 powder. Pure Ce·(SO_4_)_2_ 4H_2_O was calcined at 800 °C in the air for 6 h to form the Re2 powder. The Ce-O-S powders were calcined at 690 °C in the air and then were quickly quenched by cold nitrogen to form Re3 powder. 

### 2.3. Desulfurization and Regeneration Assessments

**Figure 1:**
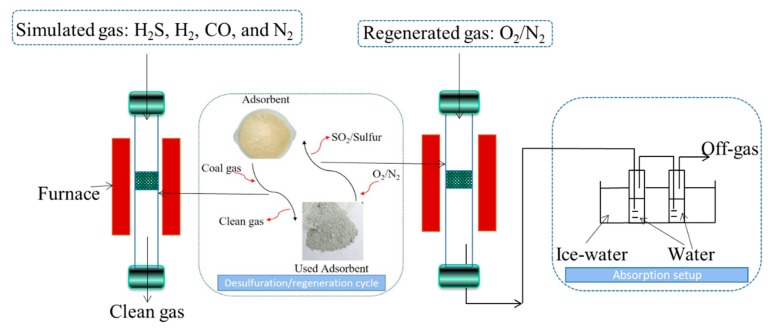
The flow diagram of desulfurization or/and regeneration system at high temperature.

The flow diagram of the desulfurization or/and regeneration system is presented in Figure 1. The sorbents were located in a quartz tube with an inner diameter of 10 mm at normal pressure using simulated hot coal gas (3000 mg/m^3^ H_2_S, 10% H_2_, 20% CO, and N_2_ balance gas). In each case, 500 mg sorbent was used for the test. The sorbents were heated to target temperature in nitrogen atmosphere at a rate of 5 °C min^−1^. Subsequently, the simulated gas mixture was introduced into the quartz tube reactor for desulfurization test. The weight hourly space velocity (WHSV) was 12 L h^−1^g^−1^, which was controlled by mass flow controllers (D07-9F/YCM, produced by Seven-star Electronics Co., Beijing, China). H_2_S concentration was detected by the iodometric method.

The outlet changes of H_2_S concentration with time can be expressed by a breakthrough curve. The breakthrough sulfur capacity (BSC) was denoted as the content of sulfur removed by the adsorbent at the breakthrough point, which can be used to assess the sorbents ability for sulfur removal. It can be evaluated by the following formula:

(1) $$\mathrm{SC}=\mathrm{WHSV}\times \frac{M_{S}}{V_{m}}\times \frac{22.4}{M_{H_{2}S}}\left[\overset{t}{\underset{0}{\int }}\left(C_{in}-C_{out}\right)dt\right]\times 10^{-4}$$

where SC is the effective sulfur capacity of adsorbent (g S/100 g adsorbent); WHSV is the weight per hour space velocity (L h^−1^ g^−1^); $M_{S}$ and $M_{H_{2}S}$ are the molar weight of sulfur (32.06 g mol^−1^) and H_2_S (34.06 g mol^−1^), respectively; $V_{m}$ is the molar volume of H_2_S at 1 atm and 25 °C (24.5 L mol^−1^); t is the desulfurization reaction time (h); $C_{in}$ is the inlet concentration of sulfur dioxide (mg/m^3^), while $C_{out}$ is the outlet concentration.

After the adsorbent desulfurization, the used sorbent was regenerated at 800 °C with a heating rate of 10 °C/min. The gas atmosphere was an air stream with a WHSV of 12 L h^−1^ g^−1^. The regeneration process was stopped until SO_2_ could not be detected. The system was flushed by N_2_ stream for 1 h after the regeneration process. Then, the new cycle process began. Each value of the sulfur capacity is the average of three measurements.

### 2.4. Sulfur Collection Test

The sulfur collection experiment was conduct in a quartz tube. Air (10 mL/min, STP) or 2% O_2_/N_2_ gas mixture (5 mL min^−1^) was introduced to the quartz tube for the regeneration of the Ce-O-S powder. The sample with different weights (250, 500, 1000, or 1500 mg) was calcined at 800 °C for 6 h in air. The off-gas was finally immersed into an adsorption setup (two adsorption bottles loaded with cold water) to collect SO_2_ or elemental S. We use classic acid–base titration to measure the acid yield. 

### 2.5. Characterizations 

The structures of all the sorbents were analyzed by X-ray diffraction (XRD, Bruker D8) equipped with Copper-Ka radiation. The scan angle (2θ) was collected from 20° to 80° with a scan rate of 5° min^−1^. X-ray photoelectron spectroscopy with an Al Kα X-ray (XPS, Perkin-Elmer model PHI 5600 system) analyzed the surface compositions of the samples. 500 mg Ce-O-S powder was heated up to 800 °C with a rate of 5 °/min in air atmosphere (10 mL min^−1^ air stream, STP), and the end-gas was tested by Fourier transform infrared spectroscopy (FTIR) (Thermo Fisher Scientific, Nicolet 6700) with scanning range from 4000 to 600 cm^−1^. The thermodynamic behaviors were conducted by thermo gravimetric analysis (TGA, NETZSCH STA 449 F3). Around 14.5 mg spent adsorbent or pure Ce(SO_4_)_2_·4H_2_O was analyzed by TGA. The sample was heated in air condition from room temperature to 900 °C with a heating rate of 8 °C/min.

## 3. Results and Discussion

### 3.1. Performance of the Sorbents 

**Figure 2:**
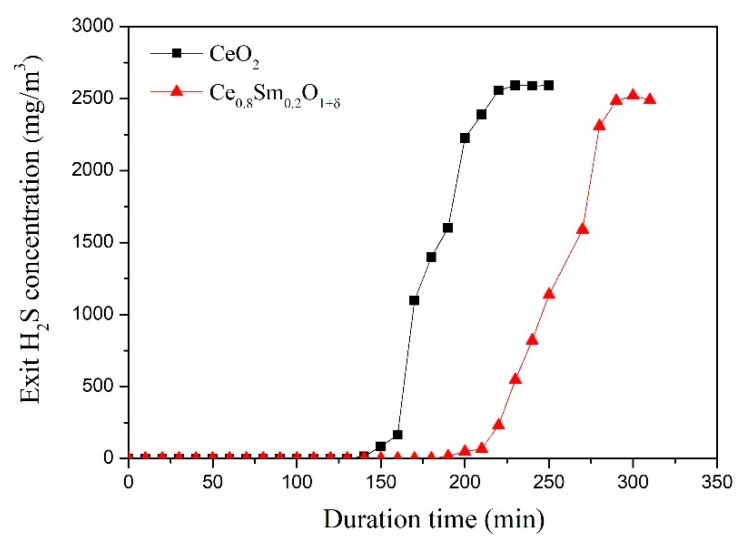
Breakthrough curves of CeO_2_ and samarium-doped cerium (SDC) sorbents (800 °C; 12 L h^−1^ g^−1^; feed: 3000 mg/m^3^ H_2_S, 10% H_2_, 20% CO, and N_2_ balance gas).

**Figure 3:**
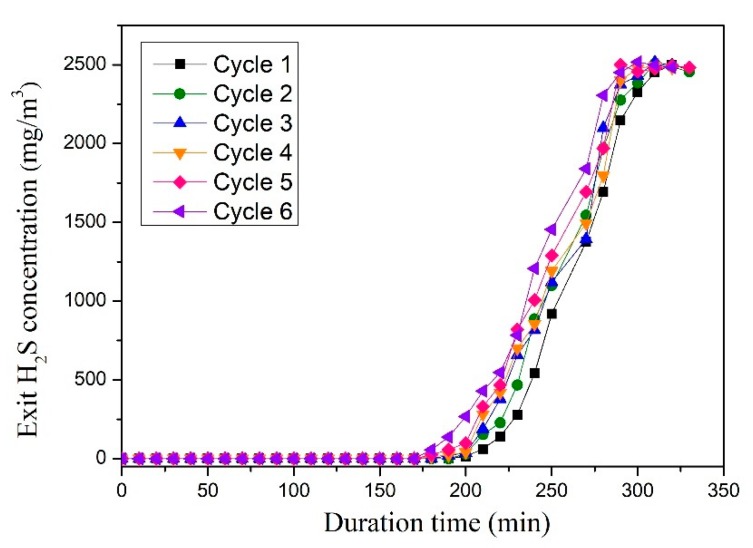
Breakthrough curves of six consecutive cycles of SDC sorbent at 800 °C (12 L h^−1^ g^−1^; feed: 3000 mg/m^3^ H_2_S, 10% H_2_, 20% CO, and N_2_ balance gas).

Figure 2 shows the H_2_S removal capacity and breakthrough curves of pure CeO_2_ and SDC sorbents at 800 °C. As shown, compared with pure CeO_2_ sorbent, SDC sorbent shows a long breakthrough time, suggesting that doping Sm can improve the sulfur adsorption capacity of CeO_2_. The maximal sulfur capacity of CeO_2_ and SDC is 7.9 and 12.1 g S/100g sorbent, respectively. Figure 3 shows the regenerated ability of the SDC sorbent at 800 °C. As shown, after six continuous cycles, the sulfur capacity of regenerated adsorbent is similar to the fresh sorbent. It can be seen that there is a slight decline in desulfurization capacity after the first cycle. The tiny loss of sulfur capacity can be ascribed to sintering of the sorbent and the active components aggregation, as a large amount of heat is released during the regeneration process [18]. The breakthrough sulfur capacity changes ranging from 10.1 to 11.9 g S/100g. In addition, the deactivation curves after the breakthrough point have a similar tendency. This suggests that SDC is a thermal-stable desulfurization sorbent. 

Table 1 shows the microstructural properties of sorbents. As shown, the BET surface area of the CeO_2_ is only 64 m^2^ g^−1^. The total pore volume and pore size values of the CeO_2_ are 0.117 cm^3^ g^−1^ and 9.86 nm, respectively. As for fresh SDC sorbent, the surface area can reach up to 271 m^2^ g^−1^, and the total pore volume is 0.362 cm^3^ g^−1^, causing a superior desulfurization performance (Figure 2). This suggests that the doped Sm plays a positive role in improving the CeO_2_ surface area. In addition, the microstructural properties of the regenerated SDC sample are also investigated. As shown, an obvious phenomenon could be found for the BET results. Basically, the surface area has a decreasing tendency, which is from 269 to 245 m^2^ g^−1^ after six cycle tests. However, for the crystallite size (D) and average pore size (P), an increasing tendency could be found, suggesting that the SDC sorbent exhibits a slight sintering phenomenon during the six regenerations process at high temperature.

### 3.2. Regeneration Mechanism of the SDC Sorbent

**Figure 4:**
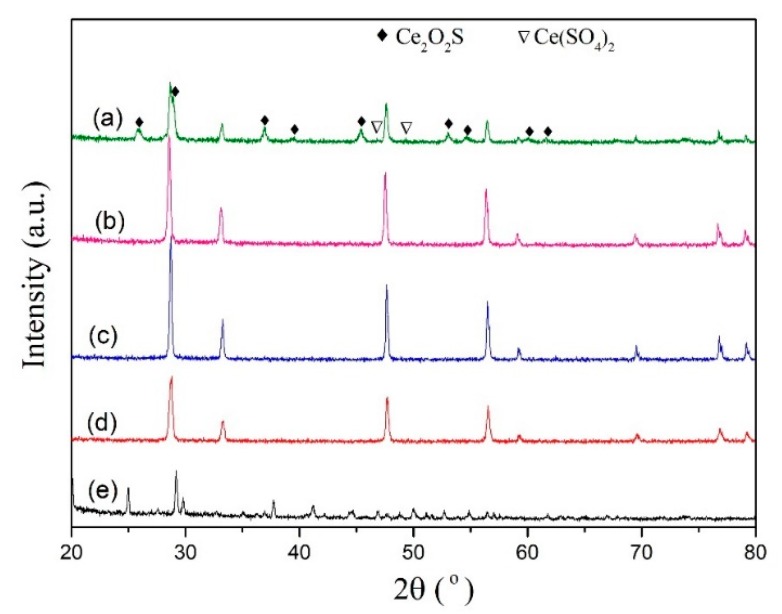
XRD patterns of (**a**) Ce-O-S powder, (**b**) Re1 powder, (**c**) fresh CeO_2_, (**d**) Re2 powder, and (**e**) fresh Ce(SO_4_)_2_·4H_2_O.

The SDC sorbents show a typical fluorite structure, and the main component of SDC is CeO_2_. Thus, the investigation of the regeneration mechanism of the SDC sorbent can be simplified into the cerium oxide regeneration according to some reports [31,32]. Figure 4 shows the XRD patterns of different powders. Ce_2_O_2_S diffraction peaks are clearly observed after desulfurization process of the SDC sorbent as shown in Figure 4a. The main sulfur-containing phase in the Ce-O-S powder is Ce_2_O_2_S phase. Apart from Ce_2_O_2_S, sulfate can also be found, which is ascribed to the transformation between the Ce_2_O_2_S and Ce(SO_4_)_2_ after H_2_O producing. The various Ce-O-S phases come from the following reactions [33]:

2CeO_2_(s) + H_2_(g) + H_2_S(g) → Ce_2_O_2_S(s) + 2H_2_O(g) (2)

2CeO_2_ + 8H_2_O + 3H_2_S → Ce_2_(SO_4_)_3_ + 11H_2_ (3)

Ce_2_(SO_4_)_3_ + 4H_2_O + H_2_S → 2Ce(SO_4_)_2_ + 5H_2_ (4)

The diffraction peak of the Re1 powder is the same with pure CeO_2_ pattern as shown in Figure 4b,c, suggesting that Ce-O-S powder could be regenerated to the CeO_2_ powder after regeneration process in air. To observe clearly, pure Ce(SO_4_)_2_ was treated by the same regeneration process. As shown in Figure 4d,e, the Re 2 powder is confirmed to be CeO_2_ phase, suggesting that Ce(SO_4_)_2_ could also be regenerated to CeO_2_ after calcination in air stream.

**Figure 5:**
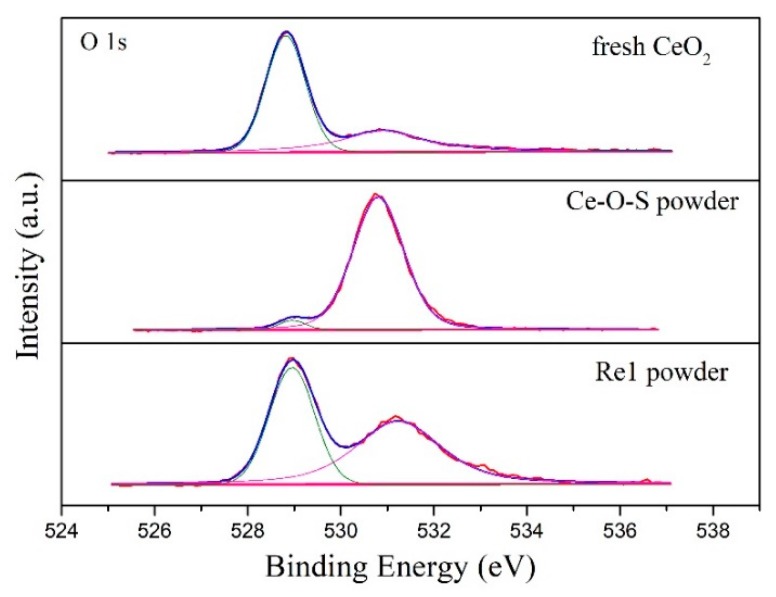
O 1s XPS spectra of pure CeO_2_, Ce-O-S powder, and Re1 powder.

**Figure 6:**
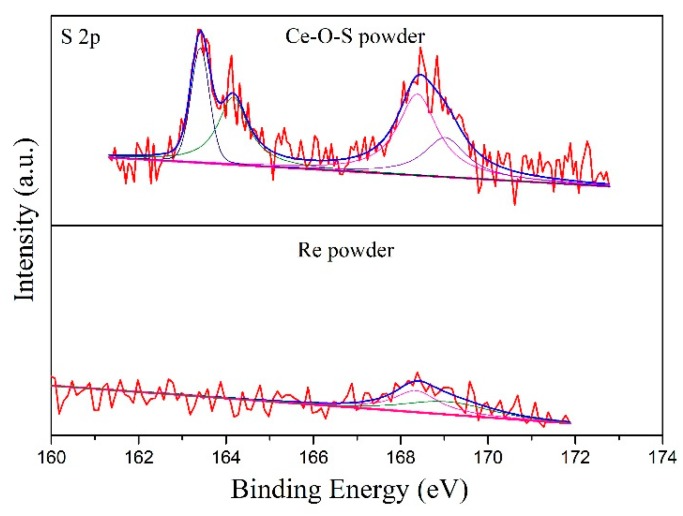
XPS spectra of S 2p of Ce-O-S powder and Re1 powder.

Figure 5 displays the XPS spectrum of O 1s of pure CeO_2_, Ce-O-S, and Re1 powder. Oxygen-related peaks located at 527.5~530.0 eV have an approached peak position that belongs to crystal lattice oxygen (O_L_). The adsorbed oxygen (O_A_) peaks are observed at 530.0~535.0 eV, in line with the literature [34]. The contents of the O_L_ of different powders are showed in Table 2. Compared with pure CeO_2_, the O_L_/(O_L_ + O_A_) ratio of the Ce-O-S powder decreases significantly, which is from 91.5% to 4.7%, accompanied by an incremental peak area of adsorbed oxygen (O_A_). This indicates that O_L_ is the active composition for the H_2_S removal. As shown in reaction (2), the O_L_ is consumed by the hydrogen sulfide and hydrogen to generate water during the desulfurization process. However, after calcination of Ce-O-S powder in air (Re1 powder), the O_L_/(O_L_ + O_A_) ratio is recovered to 75.9% (Table 2), accompanied by an incremental peak area of the O_L_. Meanwhile, the different sulfur valences are found on the surface of the Ce-O-S powder and Re1 powder, as shown in Figure 6. The representative S peaks of the Ce-O-S powder located at 166.0~173.0 eV are associated with SO_3_^2-^ and SO_4_^2-^ species, while the peaks at approximately 162.0~166.0 eV are attributed to S^2-^ species [31,35]. After calcination process in air, the representative peaks of S^2−^ of the Re1 powder disappeared, and only little sulfur (1.7%) attributed to SO_4_^2−^ is found, as shown in Table 2. Thus, the above results suggest that the CeO_2_-based adsorbent (e.g., SDC in this work) after sulfur adsorption can be regenerated by calcination in air stream. The lattice oxygen is regenerated accompanied by the removal of the sulfur.

Thermal behaviors of Ce-O-S powder and fresh Ce(SO_4_)_2_·4H_2_O were investigated by TGA. The thermal behaviors of the Ce(SO_4_)_2_·4H_2_O are generally displayed as below [36].

2Ce(SO_4_)_2_ (s) → Ce_2_O(SO_4_)_3_(s) + SO_3_(g) (5)

3Ce_2_O(SO_4_)_3_ → 2Ce_3_O_2_(SO_4_)_4_ + SO_3_(g) (6)

Ce_3_O_2_(SO_4_)_4_ → 3CeO_2_ + 4SO_3_ (7)

**Figure 7:**
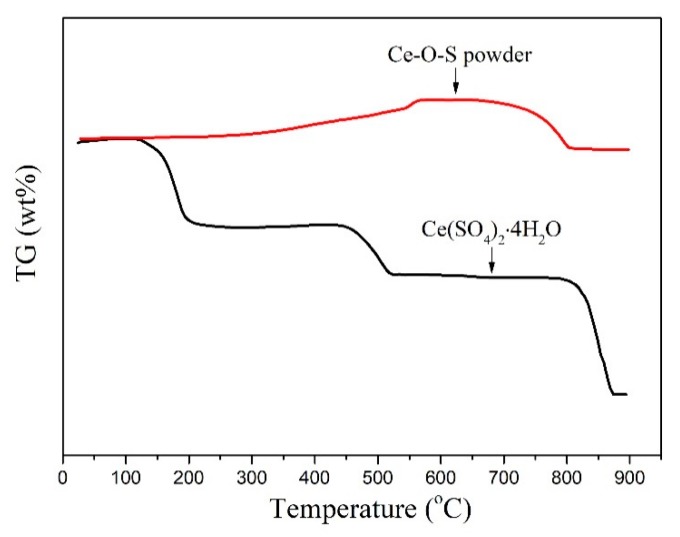
TGA curves of Ce-O-S powder and Ce(SO_4_)_2_·4H_2_O.

As shown in Figure 7, the weight loss of Ce(SO_4_)_2_·4H_2_O comes from the H_2_O removal of the endothermic powder below 300 °C, forming the crystalline Ce(SO_4_)_2_. The further weight loss of 13.5% is ascribed to the change from Ce(SO_4_)_2_ to Ce_3_O_2_(SO_4_)_4_ accompanied by the SO_3_ releasing when the temperature is heated up to 520 °C. The Ce_2_O(SO_4_)_3_ is an intermediate species during the heating process. The final products of CeO_2_ and SO_3_ are obtained above 860 °C as shown in reaction (7). However, the TGA result of the Ce-O-S powder is interesting. As shown, the endothermic process of the Ce-O-S powder displays two steps during the whole temperature-rise period. An incremental weight of around 11.3% is observed from 230 to 600 °C, and then, 11.1% weight loss can be found from 650 to 800 °C. From the results of the XRD and XPS, the sulfur species can be removed from the sorbent after calcination in the air. Thus, the weight loss (11.1%) of the Ce-O-S sample is due to the sulfur removal. It can be seen from the results that Ce_2_O_2_S is the dominant S-containing species after desulfurization. Thus, the regeneration path of the Ce_2_O_2_S is the key to the regeneration mechanism of the sorbent. However, the regenerated path of the Ce_2_O_2_S during the heating process in oxidizing atmosphere is relatively complicated [37,38,39,40]. In this study, the paths most likely are two processes, as follows.

Case 1 [37,38]:

Ce_2_O_2_S(s) + (x − 1) O_2_(g) → 2CeO_x_(s) + 1/2S_2_(g) (8)

Ce_2_O_2_S(s) + O_2_(g) → 2CeO_2_(s) + 1/2S_2_(g) (9)

Case 2 [39,40]:

Ce_2_O_2_S(s) + 2O_2_(g) → Ce_2_O_2_SO_4_(s) (10)

Ce_2_O_2_SO_4_(s) → 2 CeO_2_(s) + SO_2_(g) (11)

Considering Case-1, as shown in (8) and (9), one oxygen molecule will be captured by Ce_2_O_2_S to form CeO_2_ -molecules, accompanied by the release of the elemental S during the oxidation process. Because the oxygen molecule weight is equivalent to the weight of half S_2_ (molecular weight: O_2_ = 1/2 S_2_), the weight of the powder could be kept stable after the regeneration process. Therefore, for the path of the Case-1, the formed elemental sulfur will firstly be adsorbed on the surface of the powders below 650 °C. After 650 °C, the formed S will be desorbed with continuous flushing air and increasing temperature. For Case-2, some studies reported that Ce_2_O_2_S could combine with the oxygen to form Ce_2_O_2_SO_4_, which is relatively stable below 700 °C [40]. The Ce_2_O_2_SO_4_ will decompose into CeO_2_ and SO_2_ after the temperature is above 700 °C. Thus, Case-2 is also most likely to be the path of the Ce_2_O_2_S regeneration. 

**Figure 8:**
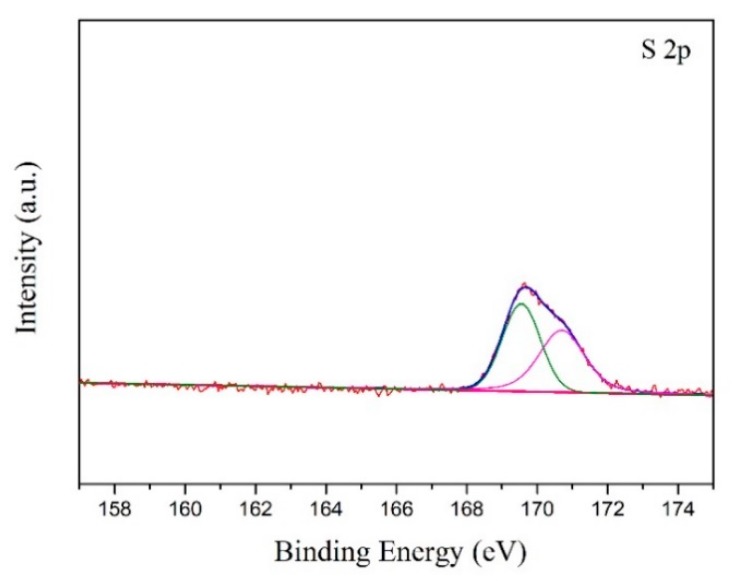
XPS spectra of S 2p of the Re3 powder.

**Figure 9:**
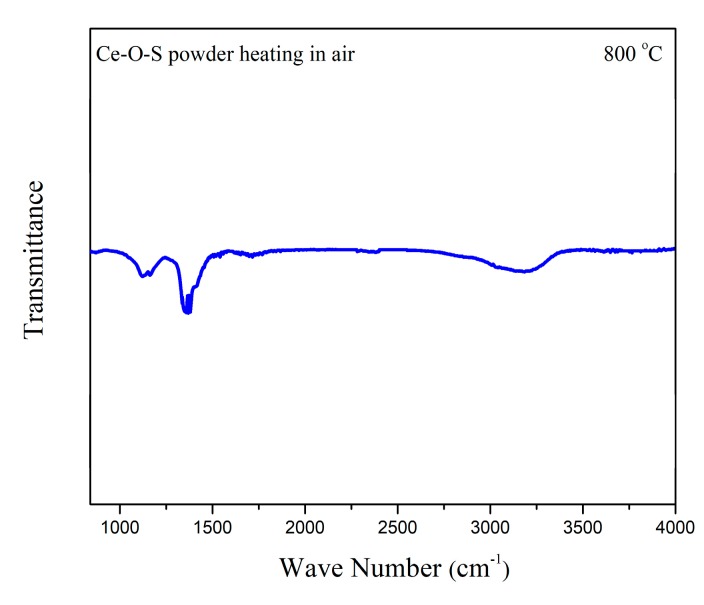
FTIR spectra of the Ce-O-S powder heating in air atmosphere (10 mL/min, STP) at 800 °C.

To confirm the regeneration route of the Ce_2_O_2_S, XPS was characterized to investigate the sulfur valence of the Re3 powder. The Ce-O-S powder was firstly heated to 690 °C in air conditions and then was fleetly cooled down to form Re3 powder. If Case-1 is the real regeneration path, the elemental sulfur will be observed on the surface of the Re3 powder. As shown in Figure 8, the S 2p spectra is close to the result of Figure 6. S^0^ was not found and only S^6+^ was observed, indicating that the regeneration path is not Case-1. Thus, Case-2 should be confirmed during the heating process. Fourier transform infrared spectroscopy (FTIR) result confirmed the speculation of Case-2. As shown in Figure 9, the peaks of SO_2_ located at 1000~1200 cm^−1^ [41,42] and the intense peaks of SO_3_ located at 1300~1500 cm^−1^ were observed [43,44]. The SO_3_ is obtained by the further oxidation of the SO_2_. Therefore, from the above results, the regeneration path of the Ce_2_O_2_S species follows Case-2, when the powder is calcined in air conditions. Figure S1 shows SEM images of the morphology of the fresh SDC (a), the surface of the fresh SDC (b), the surface of the used SDC (c), and the surface of the Re1 powder (d). As shown, the whole morphology of the fresh SDC presents a flake-like structure. The surface of the SDC is pretty smooth. However, as shown in Figure S1c, agglomerations, holes, and bubble-like structures are observed on the surface after the desulfurization process. After regeneration, the SDC surface becomes relatively smooth again and there are some particles corresponding to the active species or deciduous CeO_2_, as shown in Figure S1d. After desulfurization and regeneration, many flake-like and rectangular particles corresponding to SDC are still present, suggesting that this adsorbent has high thermal stability and regeneration capacity.

**Figure 10:**
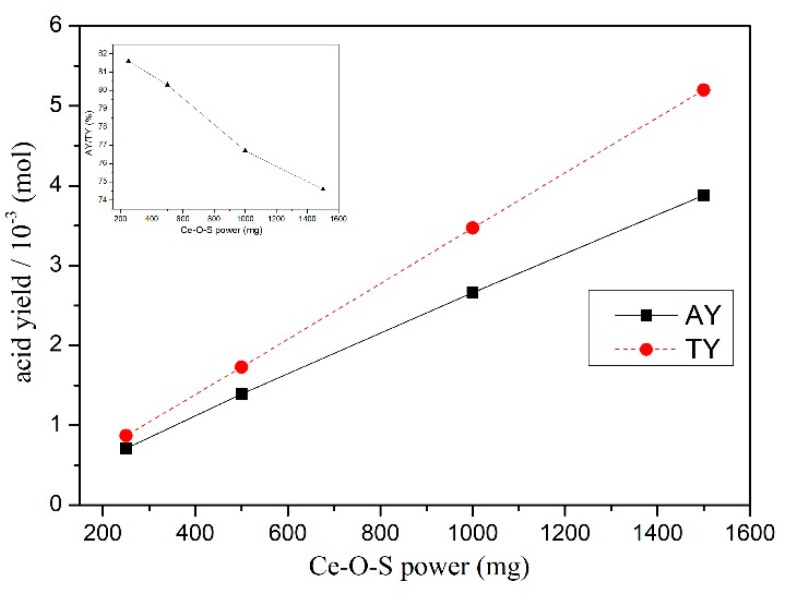
The actual yield (AY), theoretical yield (TY), and AY/TY from the Ce-O-S powder regeneration.

The gas mixture of the SO_2_ and SO_3_ can be collected as acid during the adsorbent regeneration in the air. According to the TGA result, 11.1% weight loss is attributed to the sulfur removal, and thus, theoretical yields (TY) of the acid (mol) are the same with the S loss (mol). However, the actual yields (AY) of acid can be calculated through the classical acid–base titration. Additionally, the ratio (mol%) of the AY and the consumed NaOH is 1:2. Thus, the AY/TY can be described as follows.

(12) $$\mathrm{AY}/\mathrm{TY}\;\left(\%\right)=\frac{C_{\left(NaOH\right)}\times V_{\left(NaOH\right)}\times 0.5}{\frac{m_{\left(Ce-O-S\;powder\right)}}{M_{sulfur}}\times 11.1\%}$$

where C_(NaOH)_ is the molar concentration of NaOH, V_(NaOH)_ is the consumed volume of NaOH, *m* is the weight of the Ce-O-S, and *M* is molar mass of S. Figure 10 and Table 3 show the AY, TY, and AY/TY versus the weight of the Ce-O-S powder regeneration. As shown, the AY is about 74%~82% of the theoretical yield; the AY increases with the rise of the Ce-O-S powder weight, but the AY/TY decreases. This is because Ce-O-S powder is not completely regenerated, which also can be seen from the results of Figure 6 and Table 2. 

**Figure 11:**
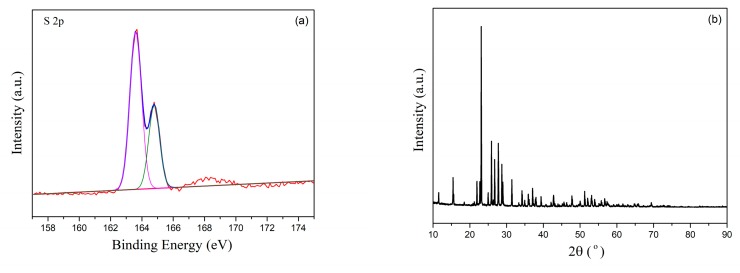
XPS spectra (**a**) and XRD pattern (**b**) of the particles after adsorbing the off-gas from the heated process of the Ce-O-S powder under a 2% O_2_/N_2_ (10 mL/min, STP) for 6 h at 800 °C.

During the investigation process, we find that elemental S can be obtained during the regeneration process in a 2% O_2_/N_2_ gas mixture. After the regeneration process of Ce-O-S powder (i.e., 2% O_2_/N_2_ (5 mL min^−1^, STP) for 6 h), the adsorption bottle precipitates some particles in the water and on the wall and bottom. Figure 11 shows the XPS and XRD result of these particles. The representative doublet peak of the S 2p located at 162~166 eV is associated with elemental sulfur [45,46], and only S^0^ valence is observed, as shown in Figure 11a. Additionally, the diffraction peaks of the particles in the XRD pattern are attributed to the elemental sulfur, as shown in Figure 11b, indicating that these precipitates are elemental S. The elemental sulfur can be precisely obtained through the regeneration path of Case 1 in a 2% O-_2_/N_2_ gas atmosphere. Some studies claimed that Ce_2_O_2_S could react with SO_2_ in high temperature (500~700 °C), resulting in the production of elemental sulfur (20% yield). However, in this study, we can obtain the elemental sulfur through precisely controlling the oxygen content during the regeneration process. This could provide a new idea for the adsorbent regeneration.

## 4. Conclusions

The SDC adsorbent showed a good sulfur removal ability and excellent regeneration capacity. The maximal sulfur capacity of SDC sorbent reaches up to 12.1 g S/100g sorbent, while the BSC of the CeO_2_ is 7.9 g S/100g sorbent. The regeneration capacity of the adsorbents occurs because the Ce-O-S species can easily be regenerated to CeO_2_ in oxidizing atmosphere. Ce_2_O_2_S is the main sulfur-containing species, and thus, the key of the regeneration mechanism of the adsorbent is the regeneration path of the Ce_2_O_2_S. There are two regeneration paths for the Ce_2_O_2_S at high temperature in O_2_/N_2_ gas mixture. The end products of the Ce_2_O_2_S-containing powder are the CeO_2_ and SO_2_ after the regeneration process in air conditions. The Ce_2_O_2_SO_4_ is an intermediate product during the heating process. However, the Ce_2_O_2_S directly generates CeO_2_ and elemental sulfur in a 2% O_2_/N_2_ gas condition.

## Figures and Tables

**Table 1:** Surface area (S), total pore volume (V), crystallite size (D), and average pore size (P) of sorbents.

Samples	S (m^2^ g^−1^)	D (nm)	V (cm^3^ g^−1^)	P (nm)
CeO_2_	64	9.1	0.117	9.86
SDC	271	5.2	0.362	5.46
SDC ^a^ (cycle 1)	269	5.7	0.347	6.53
SDC ^a^ (cycle 2)	260	6.6	0.339	6.91
SDC ^a^ (cycle 3)	256	7.2	0.341	7.45
SDC ^a^ (cycle 4)	249	7.5	0.325	7.58
SDC ^a^ (cycle 5)	251	8.0	0.317	8.16
SDC ^a^ (cycle 6)	245	8.2	0.311	8.24

^a^ regenerated SDC sample after regeneration process.

**Table 2:** XPS results of the O and S for the CeO_2_, Ce-O-S, and Re1 powders.

Samples		O 1s		S 2p
	O_L_ Position (eV)	O_A_ Position (eV)	O_L_/(O_L_ + O_A_) %	S-species Mass %
fresh CeO_2_	529.1	531.1	91.5	-
Ce-O-S powder	528.9	531.0	4.7	13.8
Re1 powder	529.0	531.1	75.9	1.7

**Table 3:** Acid yield of Ce-O-S powder following the regeneration path of Case 2.

Ce-O-S Powder (mg)	NaOH Dosage 0.05 mol/L (mL)	Actual Yield (AY) of Acid (mol)	Theoretical Yield (TY) of Acid (mol)	AY/TY (%)
250	28.3	0.71 × 10^−3^	0.87 × 10^−3^	81.6
500	55.6	1.39 × 10^−3^	1.73 × 10^−3^	80.1
1000	106.4	2.66 × 10^−3^	3.47 × 10^−3^	76.7
1500	155.2	3.88 × 10^−3^	5.20 × 10^−3^	74.6

## Supplementary Materials

The following are available online at https://www.mdpi.com/1996-1944/13/5/1225/s1, Figure S1: SEM images of the morphology of the fresh SDC (a), the surface of the fresh SDC (b), the surface of the used SDC (c) and the surface of the Re1 powder (d).
