# Supplementary material for: Regeneration Mechanism of Sulfur Absorption Via Samarium-doped Cerium Adsorbents in the Gas Atmosphere of O2/N2
Source: Materials (Basel). 2020 Mar 9;13(5):1225. doi: 10.3390/ma13051225 (PMC7085105; doi:10.3390/ma13051225)
Supplement: Supplementary file 1 [file materials-13-01225-s001.pdf]

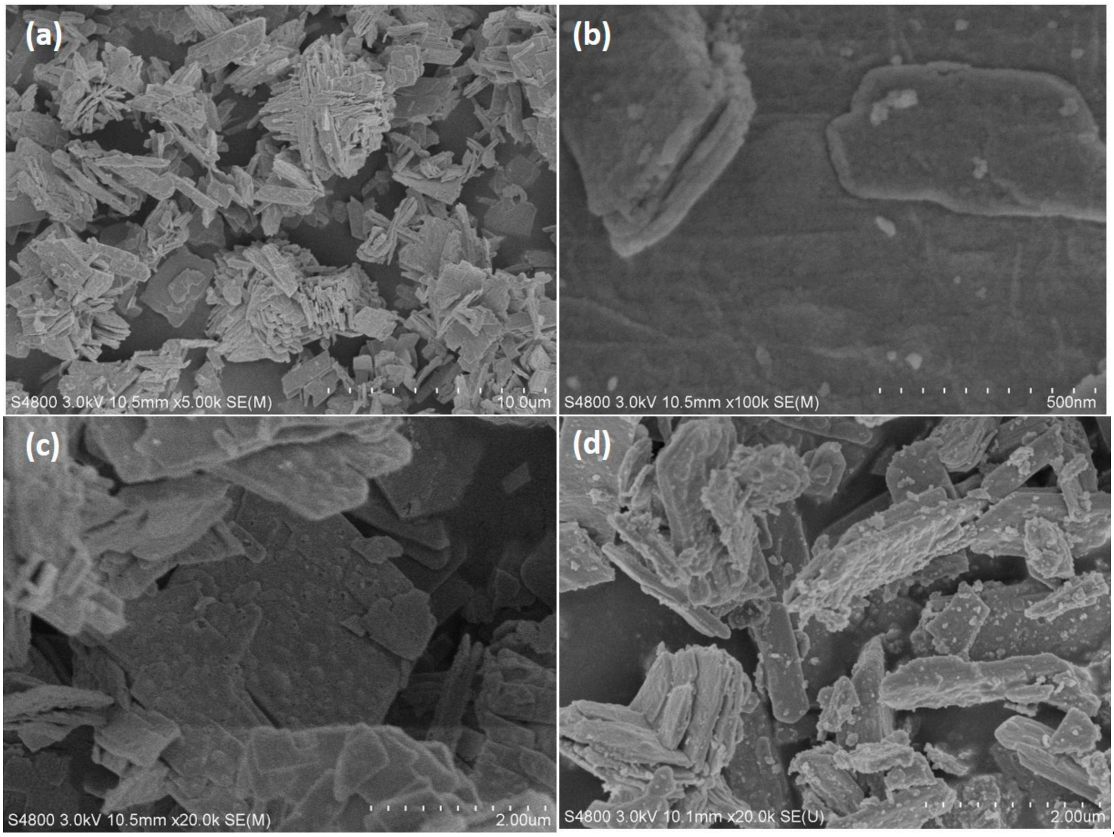

Figure S1. SEM images of the morphology of the fresh SDC (**a**), the surface of the fresh SDC (**b**), the surface of the used SDC (**c**) and the surface of the Re1 powder (**d**).
